# Towards computational reproducibility: researcher perspectives on the use and sharing of software

**DOI:** 10.7717/peerj-cs.163

**Published:** 2018-09-17

**Authors:** Yasmin AlNoamany, John A. Borghi

**Affiliations:** 1 University of California, Berkeley, CA, United States of America; 2 California Digital Library, Oakland, CA, United States of America

**Keywords:** Software sustainability, Reproducibility, Research software, Code, Finding software, Sharing software

## Abstract

Research software, which includes both source code and executables used as part of the research process, presents a significant challenge for efforts aimed at ensuring reproducibility. In order to inform such efforts, we conducted a survey to better understand the characteristics of research software as well as how it is created, used, and shared by researchers. Based on the responses of 215 participants, representing a range of research disciplines, we found that researchers create, use, and share software in a wide variety of forms for a wide variety of purposes, including data collection, data analysis, data visualization, data cleaning and organization, and automation. More participants indicated that they use open source software than commercial software. While a relatively small number of programming languages (e.g., Python, R, JavaScript, C++, MATLAB) are used by a large number, there is a long tail of languages used by relatively few. Between-group comparisons revealed that significantly more participants from computer science write source code and create executables than participants from other disciplines. Differences between researchers from computer science and other disciplines related to the knowledge of best practices of software creation and sharing were not statistically significant. While many participants indicated that they draw a distinction between the sharing and preservation of software, related practices and perceptions were often not aligned with those of the broader scholarly communications community.

## Introduction

Research software is an important consideration when addressing concerns related to reproducibility (Hong, 2011; Hong, 2014; Stodden, Leisch & Peng, 2014; Goble, 2014). Effective management and sharing of software saves time, increases transparency, and advances science (Prlić & Procter, 2012). At present, there are several converging efforts to ensure that software is positioned as a “first class” research object that is maintained, assessed, and cited in a similar fashion as scholarly publications (e.g., NIH, 2016; Katz et al., 2013; Ram et al., 2017; Crouch et al., 2013). However, while there is a burgeoning literature exploring the activities of researchers in relation to materials like data (Tenopir et al., 2015; Monteith, McGregor & Ingram, 2014; Kim & Stanton, 2016), those related to software have received less attention. Specifically, we have been unable to find a study that thoroughly examines how researchers use, share and value their software.

In this paper, we report the results of a survey designed to capture researcher practices and perceptions related to software. Survey questions addressed a variety of topics including:

1. What are the characteristics of research software?
2. How do researchers use software?
3. To what extent do current practices related to software align with those related to reproducibility?
4. How do researchers share software?
5. How do researchers preserve software?

After filtering, 215 researchers participated in our survey. Overall, our results demonstrate that researchers create software using a wide variety of programming languages, use software for a wide variety of purposes, have adopted some—but not all—practices recommended to address reproducibility, often share software outside of traditional scholarly communication channels, and generally do not actively preserve their software. Participants from computer science reported that they write source code and create executables significantly more than participants from other disciplines. However, other between-group comparisons largely did not reach statistical significance.

In the following sections, we provide a more detailed description of our findings. We start with an overview of the related literature (‘Related Work’) then a description of our survey instrument (‘Methods’) and the demographic characteristics of our participants (‘Participant Demographics’). In ‘Characteristics and Use of Research Software’, we describe our findings related to the characteristics of research software and its usage. Responses to questions involving reproducibility-related practices are detailed in ‘Reproducibility-related Practices’. ‘Sharing and Preservation of the Research Software’ outlines the responses to questions related to software sharing and preservation. We discuss the implications of our findings in ‘Discussion’. Finally, ‘Conclusions and Future Work’ concludes our findings and contains a discussion of future work.

## Related Work

While there is an emerging body of research examining researcher practices, perceptions, and priorities for products like data (Fecher, Friesike & Hebing, 2015; Kratz & Strasser, 2015; Tenopir et al., 2011; Tenopir et al., 2015), work related to software has often focused on how it is found, adopted, and credited (Howison & Bullard, 2015b; Hucka & Graham, 2018; Joppa et al., 2013). For example, research examining software reuse demonstrates that the most common difficulty for users looking for software is a lack of documentation and that finding software is a difficult task even within technology companies (Sadowski, Stolee & Elbaum, 2015). However, as software is increasingly central to the research process (Borgman, Wallis & Mayernik, 2012), understanding its characteristics, its uses, and the related practices and perceptions of researchers is an essential component of addressing reproducibility.

The term “reproducibility” has been applied to a variety of efforts aimed at addressing the misalignment between good research practices, including those emphasizing transparency and methodological rigor, and the academic reward system, which generally emphasizes the publication of novel and positive results (Nosek, Spies & Motyl, 2012; Munafò et al., 2017). Attempts to provide a cohesive lexicon for describing reproducibility-related activities are described elsewhere (Goodman, Fanelli & Ioannidis, 2016) but *computational reproducibility* generally refers to the description and sharing of software tools and data in such a manner as to enable their use and evaluation by others (Stodden, Guo & Ma, 2013). Efforts aimed at fostering computational reproducibility are often focused on the sharing of source code but may also include the establishment of best practice guidelines related to how software tools are described, cited, and licensed (e.g., Stodden et al., 2016).

There have been numerous calls urging researchers to more thoroughly describe and share their software (Barnes, 2010; Ince, Hatton & Graham-Cumming, 2012; Joppa et al., 2013; Morin et al., 2012). Such calls are increasingly backed by mandates from funding agencies. For example, the Wellcome Trust now expects that grant recipients make available “any original software that is required to view datasets or to replicate analyses” (Wellcome, 2017). In parallel, a myriad of guidelines, organizations, and tools have emerged to help researchers address issues related to their software. Software-related best practices have been outlined for both individuals working in specific research disciplines (Eglen et al., 2017; Marwick, 2017) and for the research community in general (e.g., Piccolo & Frampton, 2016; Sandve et al., 2013; Jimenez et al., 2017). In general, such best practice documents focus on the importance of concepts such as proper documentation and version control in ensuring that code is shared in a way that facilitates computational reproducibility. In contrast, the focus of community organizations such as The Carpentries (Wilson, 2006; Teal et al., 2015) and the Software Sustainability Institute (Crouch et al., 2013) is training researchers to better develop, use, and maintain software tools. Bridging the perspectives of stakeholders focused on encouraging best practices in sharing software and those focused on educating researchers in its creation and use are organizations such as Force11, who have published guidelines for describing and citing software in the scholarly literature (Smith, Katz & Niemeyer, 2016).

Complementing best practices and educational materials, a variety of tools have been developed to facilitate computational reproducibility. For example, literate programming tools such as Jupyter notebooks (Perez & Granger, 2007) allow researchers to combine data, code, comments, and outputs (e.g., figures and tables) in a human-readable fashion, while packaging and containerization platforms such as ReproZip (Chirigati, Shasha & Freire, 2013) and Docker (Boettiger, 2015) enable the tracking, bundling, and sharing of all of the software libraries and dependencies associated with a research project. Through their integration with GitHub (https://github.com/), services like Figshare (https://figshare.com/) and Zenodo (https://zenodo.org/) allow researchers to deposit, archive, and receive persistent identifiers for their software.

As is evident in the above description, reproducibility-related efforts involving software often, but not always, overlap with those related to data. However, software presents a number of unique challenges compared to data and other research products (Chassanoff et al., 2018). Even defining the bounds of the term “software” is challenging. For example, the National Institute of Standards and Technology (NIST) defines software as “Computer programs and associated data that may be dynamically written or modified during execution.” (Kissel et al., 2011), a definition that is as recursive as it is potentially confusing for researchers without a background in computer science or software development. Software involves highly interdependent source and binary components that are sensitive to changes in operating environment and are difficult to track (Thain, Ivie & Meng, 2015). Evaluating the validity and reliability of software often requires inspecting source code, which is not possible when proprietary licenses are applied (Morin, Urban & Sliz, 2012; Stodden, 2009). Even when source code is technically available, important information about versions, parameters, and runtime environments is often missing from the scholarly record (Howison & Bullard, 2015b; Pan, Yan & Hua, 2016; Stodden, Guo & Ma, 2013). Seemingly small alterations, even for well described and openly available software tools, can lead to significant effects on analytical outputs (McCarthy et al., 2014), a problem exacerbated by the fact that researchers often have minimal formal training in software development practices (Hannay et al., 2009; Joppa et al., 2013; Prabhu et al., 2011). The iterative and collaborative nature of software development also means that it does not fit easily within existing academic incentive structures (Hafer & Kirkpatrick, 2009; Howison & Herbsleb, 2011; Howison & Herbsleb, 2013), which makes it difficult to create incentives to follow best practice.

Beyond the communities actively using it as part of the research process, software is also a growing concern among research service providers. For example, services related to software preservation (e.g., Rios, 2016) and emulation (e.g., Cochrane, Tilbury & Stobbe, 2018) have been explored by academic libraries and promoting specific tools and best practices related to software is central to approaches that can be broadly defined as “reproducibility librarianship” (Sayre & Riegelman, 2018; Steeves, 2017). Through workshops, often facilitated through organizations such as The Carpentries (Wilson, 2006; Teal et al., 2015), many academic libraries have also begun to provide guidance and training to researchers looking to create and use software tools. However, these activities remain relatively nascent and it is presently unclear what a mature set of services related to research software and computational reproducibility might look like. By identifying the characteristics of research software, its uses, and elucidating the related practices and perceptions of researchers, we hope to establish a benchmark that can be applied to inform the development of such services in the future. We also hope that our survey instrument, which we purposely designed to assess a wide picture of how researchers use and share their software, will be reused or adapted by research service providers and digital libraries as they design or refine services related to research software and computational reproducibility.

## Methods

In order to understand researcher practices and perceptions related to software and computational reproducibility, we designed and disseminated an online survey via the Qualtrics platform (http://www.qualtrics.com). The survey was advertised through blog posts, social media, and data- and research-related e-mailing lists and listservs. Because we cannot know how many potential participants saw the survey and chose not to respond, we were unable to calculate a response rate. In ‘Participant Demographics’, we detail the demographics of the survey’s participants. Though these participants represent a broad range of research areas, it is likely that our responses were influenced by our use of computer science and neuroscience-focused e-mail lists.

All study materials and procedures were approved by the University of California Berkeley Committee for Protection of Human Subjects and Office for the Protection of Human Subjects (protocol ID 2016-11-9358). The full text of the survey can be found in the supplementary materials. Before beginning the survey, participants were required to read and give their informed consent to participate. After reading the informed consent form (see survey), participants indicated their consent by checking a box. Information from participants who did not check this box was removed from all subsequent analyses. An anonymized version of our survey results (AlNoamany & Borghi, 2018a), in which information that could be potentially used to identify study participants (e.g., institution), as well as the code we used for the analysis (AlNoamany & Borghi, 2018b) are available through the University of California’s data publication platform Dash and Zenodo, receptively. Study materials are also available on GitHub (https://github.com/yasmina85/swcuration).

### Survey design

The survey was developed to capture a broad range of information about how researchers use, share, and value their software. The final survey instrument consisted of 56 questions (53 multiple choice, three open response), divided into four sections. In order, the sections focused on:

1. Demographics: included questions related the participant’s research discipline, role, degree, age, institution, and funding sources (seven questions).
2. Characteristics of research software: included questions related to how the participants use software and the characteristics of their software (17 questions).
3. Software sharing practices: included questions related to how participants make their software available to others (18 questions).
4. How researchers assign value to software (14 questions).

Because only ‘Related Work’ and ‘Methods’ addressed topics related to computational reproducibility, this paper is focused on responses to questions in the first three sections. Future work will further delineate how researchers value software.

To the extent possible, survey questions that included a set of predefined responses drew upon existing data on the characteristics of research software and activities related to its use and sharing. For example, the choices for the question “Which programming language(s) do you use for writing code?” were partially based on a 2016 Stack Overflow survey (Stack Overflow, 2017) while the choices for the question “How have you cited a piece of code or software?” drew upon research into the visibility of software in the scholarly literature (Howison & Bullard, 2015a). For the non-multiple choice questions, we have used a selection of responses throughout this paper in order to illustrate trends observed in our quantitative data.

Because we hypothesized that study participants would come to our survey with different levels of knowledge about software development practices and terminology, we included a brief list of definitions in our survey for terms like “source code”, “executable”, and “open source software” that participants could refer back to at any time. Participants were also not required to answer every question in order to proceed through the survey.

### Filtering and exclusion criteria

We collected 330 responses to our online survey from late January to early April of 2017. We excluded participants who started the survey but did not answer questions beyond the demographic section, resulting in 215 participants in our final dataset. Though the majority of our participants indicated that they were from academia (Table 1), we did not exclude any participant due to institution type because of the possibility that participants could be affiliated with an academic or research program while conducting work in another sector. Institution names and disciplines were canonicalized (e.g., ‘UCB’ and ‘uc berkeley’ were mapped to UC Berkeley).

**Table 1:** Demographic breakdown for study participants.

	Count	Percentage		Count	Percentage
**Discipline**			**Institution**		
Computer Science	39	18.3%	Academic: Research Focused	164	77.0%
Biology	29	13.6%	Academic: Teaching Focused	22	10.3%
Psychology	28	13.1%	Government	13	6.1%
Engineering	13	6.1%	Nonprofit	7	3.3%
Interdisciplinary Programs	12	5.6%	Academic: Medical School	3	1.4%
Mathematics	12	5.6%	Commercial	2	0.9%
Physics	12	5.6%	Other	2	0.9%
Earth Science	9	4.2%	**Role**		
Library Sciences	9	4.2%	Graduate Student	67	31.5%
Social Sciences	9	4.2%	Postdoc	38	17.8%
others	41	19.20%	Research Faculty	35	16.4%
**Highest degree**			Staff	29	13.6%
Doctorate	110	51.9%	Principal Investigator	25	11.7%
Masters	72	34.0%	Research Assistant	10	4.7%
Bachelors	26	12.3%	Undergraduate Student	2	0.9%
High school	3	1.4%	Research	1	0.5%
Professional degree	1	0.5%	Other	6	2.8%

Because computer science had the highest representation in our sample and our assumptions that researchers in computer science would be the most likely to receive formal training in software related practices, between-group comparison were made between researchers from computer science and those from other disciplines.

## Participant Demographics

We asked participants about their age, professional degrees, professional title (or role) and institutional affiliation, institution type, and the sources of funding. The majority of these questions were multiple choice with an option for an open response upon selecting “Other”.

The mean and median age of our participants were 35.8 and 33, respectively. Reflecting the ubiquity of software within the research enterprise, participants were drawn from a wide variety of research disciplines, institution types, and roles. As shown in Table 1, the most represented disciplines in our sample were computer science, biology, and psychology. The majority of our participants were drawn from 129 different research-focused academic institutions (including 12% out of 215 researchers from UC Berkeley). Table 1 also shows that participants had a range of degrees and roles, with the most common being doctorate (51.9%, *N* = 215) and graduate student (31.5%, *N* = 215), respectively. In terms of funding, the most common responses were the National Science Foundation (NSF) (16.7%, *N* = 215) and the National Institutes of Health (7.0%, *N* = 215).

## Characteristics and Use of Research Software

In this section, we describe responses to questions related to the creation and use of source code and executables.

### Source code and executables

We asked participants about the generation and use of source code and executables: do you write source code? Do you use source code written by others? Do you create executables? Do you use executables created by others? We found that 84.2% out of 215 responding participants write source code and 89.8% out of 215 use source code written by others while 53.7% out of 214 create executables and 80.4% out of 214 use executables written by others.

**Figure 1:**
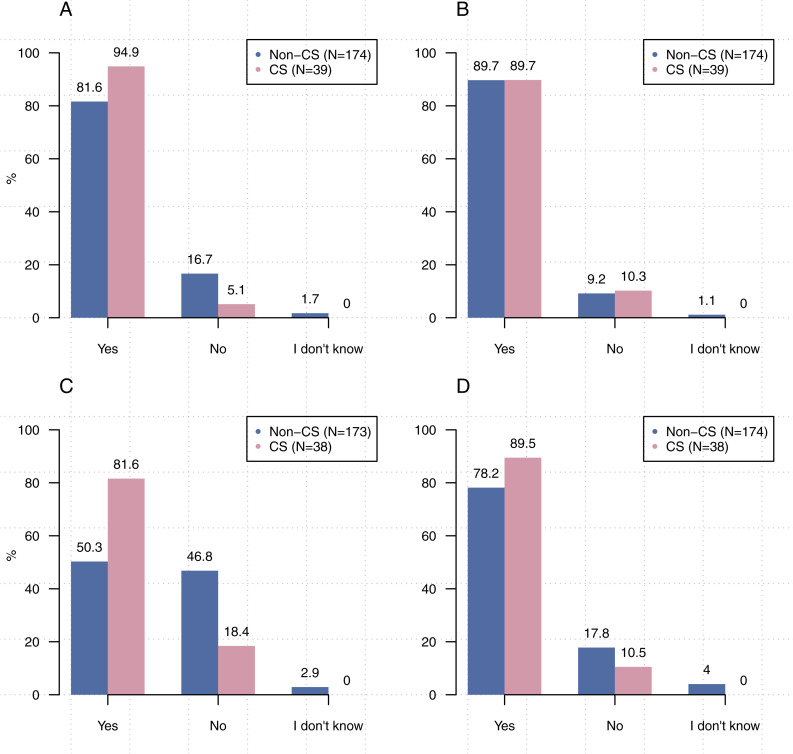
Significantly more participants from computer science stated that they write source code, create executables, and use executables created by others than participants from other disciplines. (A) Do you write source code? (B) Do you use source code written by others? (C) Do you create executables? (D) Do you use executables created by others?

Figure 1 shows that participants from computer science were significantly more likely to write source code [*χ*^2^2, (*N* = 213) =8.93,  *p* < 0 .05], create executables [*χ*^2^, (2, *N* = 211) = 22.67, *p* < 0.00001], and use executables created by others [*χ*^2^2, (*N* = 212) =6.66, *p* < 0 .05] than participants from other disciplines. Comparisons related to the use of others’ source code did not reach statistical significance [*χ*^2^2, (*N* = 213) =1.21,  *p* = 0 .55].

**Figure 2:**
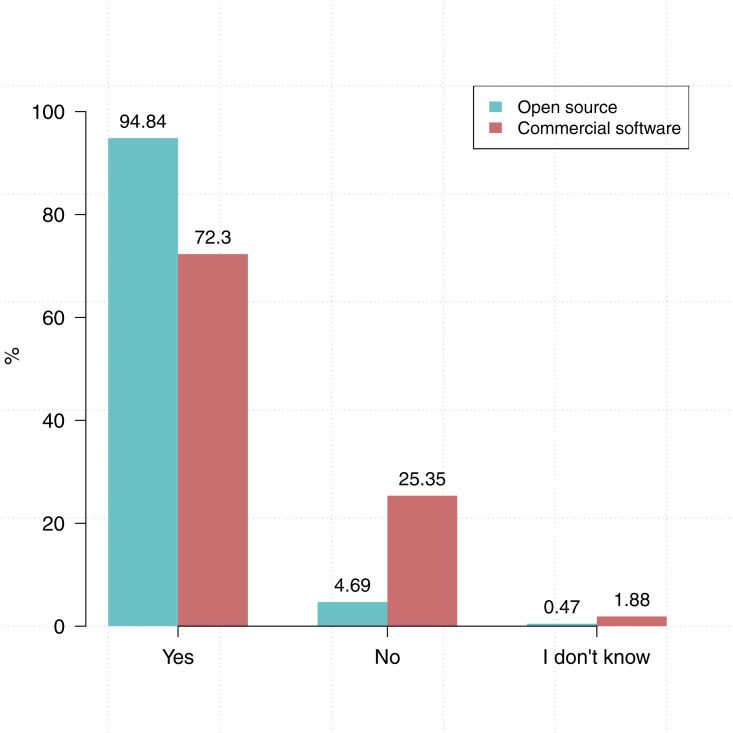
Researchers tends to use open source software (*N* = 213) more than commercial software (*N* = 212).

We also asked participants about the type of software they use: do you use commercial software in the course of your research? Do you use open source software in the course of your research? As shown in Fig. 2 more participants indicated that they use open source software (94.9%, *N* = 213) than commercial software (72.8%, *N* = 212).

### Programming languages

In order to quantify the breadth of programming languages used in a research setting, we asked participants about the languages they use when writing their own code. We presented participants with 13 languages and also provided “other” and “not applicable” as options. The 3.7% of responding participants who chose the “not applicable” option were excluded from subsequent analysis. Reflecting the complexity of defining software, our results include a mix of programming, markup, and query languages.

Table 2 shows the top ten languages, which together account for 92.6% of languages selected. The top used languages in our sample were Python, R, Javascript, C + +, MATLAB, Java, C, PHP, and Perl. Python and R were the most used languages, selected by 67.0% and 59.2% of 206 participants, respectively. For the most part, these results are in line with previous findings by (Hucka & Graham, 2018) and also match those of a recent study from Stack Overflow (Stack Overflow, 2017).

**Table 2:** The top 10 programming languages used by the researchers in our sample. A total of 206 participants answered this question. Together these languages represent 92.6% of the languages selected. Note that participants could choose more than one language.

Language	Python	R	SQL	Javascript	C+ +	MATLAB	Java	C	PHP	PERL
Selection	138	122	60	57	54	45	35	25	25	21
Percentage	67.0%	59.2%	29.1%	27.7%	26.2%	21.8%	17.0%	12.1%	12.1%	10.2%

In total, 52 different languages were chosen, with the most common responses outside of the top ten being Ruby, C#, ASP, SAS, XML, XQuery, and Julia. Quantitatively measuring the use programming languages in academic research is difficult due to the variability of reporting practices (Howison & Bullard, 2015a), but our results are largely in line the rapid ascent of R and Python as tools for data science. When participants indicated that they use multiple programming languages, the most common combinations were Python and R. Python and R were chosen together by 3.9% of 206 participants, and they are chosen together with other languages by 34.0% of participants. The second most common combinations were C++ and Python (2.9% of 206 participants) and Matlab, Python and R (2.9%, *N* = 206).

### Use of research software

Previous scholarship (e.g., Borgman, Wallis & Mayernik, 2012) has indicated that researchers use software for a wide variety of purposes. To examine the purposes of research software, we asked participants about how they use their code or software. This question allowed them to choose multiple answers from a suggested list or input other answers.

**Figure 3:**
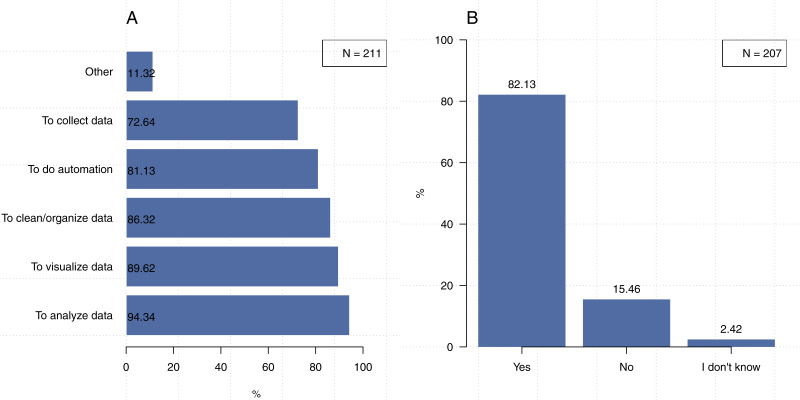
The purpose of using research software. Note that the first question could be answered with more than one choice. (A) How do you use code or software? (B) Have you ever repurposed your code or software?

Figure 3A shows that our participants use software primarily to analyze data, visualize data, clean and organize data, automate their work, and collect data. A total of 118 participants (55.7% out of 211 participants) responded that they use software for all five purposes. “Other” responses included running simulations, building models, researching algorithms, testing methods, writing compilers, and sharing and publishing data.

**Figure 4:**
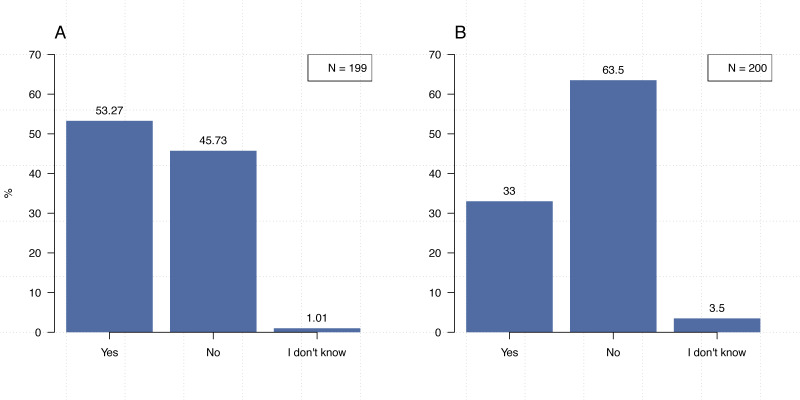
Consistency of programming languages within research groups. (A) Do you write code collaboratively? (B) Does everyone in your lab or research group write code using the same programming language(s)?

In addition to examining their use of code, we also inquired about how our participants repurpose research software. The majority (82%, *N* = 207) indicated that they repurpose code (i.e., using it for purposes other than the one for which it was originally created), see Fig. 3A. Though previous work on the reuse of research software has often, though certainly not exclusively, been focused on issues such licensing, review of code, and user awareness (e.g., Joppa et al., 2013; Morin, Urban & Sliz, 2012). These results demonstrate the need for the establishment of best practices (or good enough practices—e.g., Wilson et al., 2017) for code similar to those related to research data. After all, even a researcher who is reusing their own code, may need to refer back to relevant comments or documentation. Furthermore, though 53.3% (*N* = 199) of our participants stated that they write code collaboratively (Fig. 4A), only 33% (*N* = 200) indicated that everyone in their lab uses the same programming languages (Fig. 4B). Thus, the establishment of best practices is necessary to ensure that different team members can properly use and build upon code written in different languages for different purposes.

In an open response question, we asked participants to describe, in their own words, how they use software or code. Here, again, participants indicated that they use software for a wide variety of purposes. One participant summed the relationship between software and their research succinctly as “I use software for stimulus presentation, data acquisition, and data analysis and visualization—basically my entire research is run via computers (and thus code).” Similarly, another participant described the application of software within the field of computer science: “As a computer scientist, almost every aspect of my research from grant proposal to collecting data to analyzing data to writing up my results involves software. I write software. I use software my collaborators or students write as well as open source and commercial software.”

## Reproducibility-Related Practices

**Figure 5:**
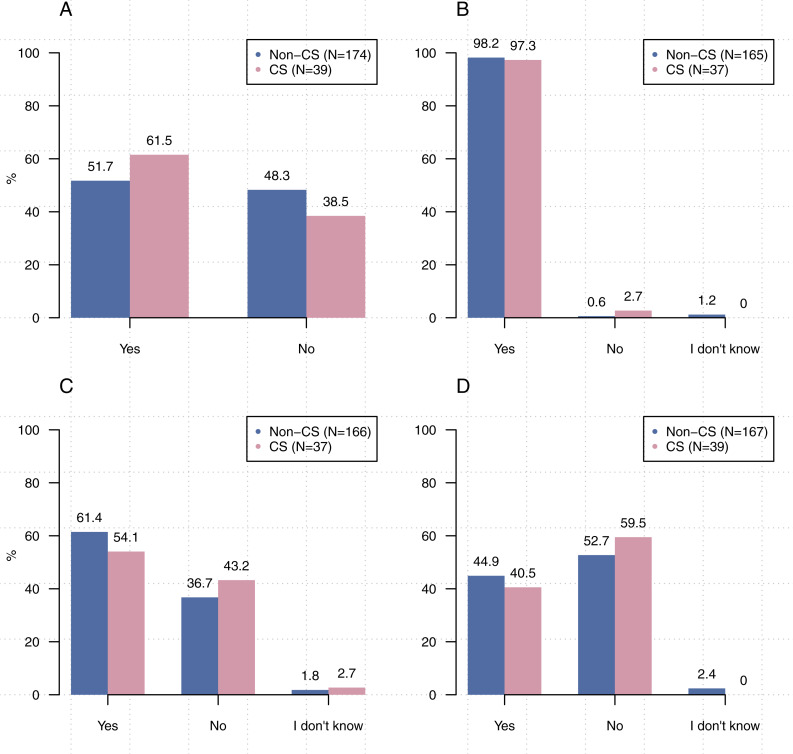
Reproducibility practices in research. (A) Have you received training in coding conventions or best practices? (B) Do you include comments in your code? (C) Do you generate documentation for your code? (D) Do you write code using a notebook?

**Figure 6:**
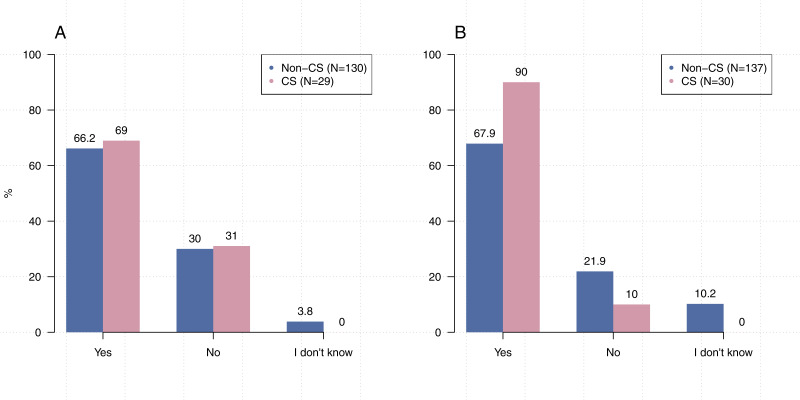
CS researchers tend to provide information about dependencies more than other disciplines. (A) When you share your code or software, do you share it alongside related files (e.g., datasets)? (B) When you share your code or software, do you provide information about dependencies?

To understand how the practices of our participants align with those related to computational reproducibility, we asked a number of questions about adding comments to source code, generating documentation, communicating information about dependencies, and using “notebook” applications such as Jupyter. We also asked about awareness of coding conventions and best practices. The results of these questions are shown in Figs. 5 and 6.

In line with previous research (Hannay et al., 2009; Joppa et al., 2013; Prabhu et al., 2011), only 53.4% (*N* = 214) of our participants indicated that they have received formal training in coding conventions or best practices. At the same time, we found that many participants actually employ practices that are commonly cited for establishing computational reproducibility. For example, when asked “Do you include comments in your code?” and “When you share your code or software, do you provide information about dependencies?” the majority of participants (98.0%, *N* = 202, 72.2%, *N* = 167) indicated that they include comments and provide information about dependencies, respectively. However, substantially fewer participants indicated that they employ other practices such as generating documentation (60.0%, *N* = 205). While electronic lab notebooks have been cited as a tool for ensuring reproducibility (Kluyver et al., 2016), only 43.6% (*N* = 206) of our participants indicated that they use them to write code.

Comparisons of responses by discipline or location were insignificant even, surprisingly, on questions related to receiving training in coding conventions or coding best practices; computer science researchers versus others (Figs. 5 and 6): [*χ*^2^1, (*N* = 213) =1.58,  *p* = 0 .21], UC Berkeley researchers versus others: [*χ*^2^2, (*N* = 200) =0.00, *p* = 1 .00]. The lone exception was in providing information about dependencies (Fig. 6B). Significantly more respondents from computer science reported that they include information about dependencies when they share their code than participants from other disciplines [*χ*^2^2, (*N* = 167) =17.755,  *p* < 0 .001].

## Sharing and Preservation of the Research Software

Making materials available for others to evaluate, use, and build upon is an essential component of ensuring reproducibility. Much of the previous work examining the sharing of research software has focused on the degree to which software is cited and described irregularly in the scholarly literature (Howison & Bullard, 2015a; Smith, Katz & Niemeyer, 2016) and the relationship between code sharing and research impact (Vandewalle, 2012). In order to gain a greater understanding of how sharing practices relate to reproducibility, we asked our participants a variety of questions about how they share, find, and preserve software.

### Sharing research software

#### Sharing practices

**Figure 7:**
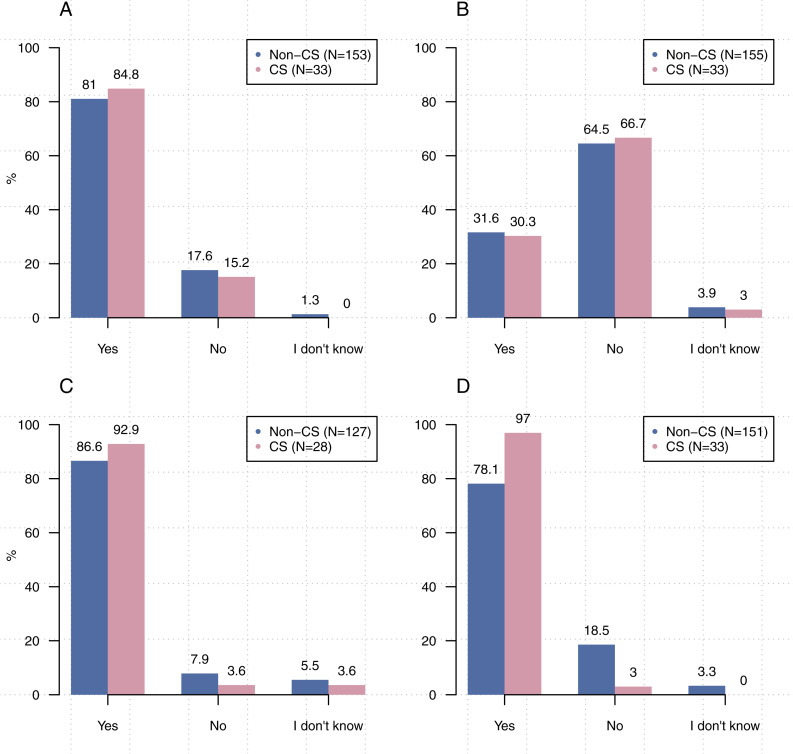
Practices of code sharing. (A) Do you share the code or software created as part of your research? (B) Is there any reason your code or software could not be shared? (C) If you make a change to your code, do you share a new version? (D) Do you use a version control system (e.g., Git)?

While only half (50.5%, *N* = 198) of our participants indicated that they were aware of related community standards of software sharing in their field or discipline, the majority indicated that they share software as part of the research process (computer science: 84.9%, other disciplines: 81.1% for *N* = 186) (Fig. 7). Of 188 participants, 31% indicated that there were reasons their software could not be shared Fig. 7B. The most commonly cited restrictions on sharing were the inclusion of sensitive data, intellectual property concerns, and the time needed to prepare code for sharing. These results are similar to those of commonly found when researchers are asked why they cannot share data (Tenopir et al., 2011). Comparisons between computer science and other disciplines on the sharing of code were not statistically significant [*χ*^2^(2, *N* = 186) = 1.5842, *p* > 0 .4529].

We checked if participants share new versions of their code and found that 87.8% (*N* = 155) do so. We also asked participants “Do you use a version control system (e.g., Git, SVN)?” and found that 81.1% (*N* = 184) indicated that they use version control system. A between-group comparison related to the sharing of new versions was not statistically significant [CS vs non-CS: *χ*^2^(2, *N* = 155) = 2.2, *p* > 0 .05] (Fig. 7C), however significantly more participants from computer science indicated that they share their code via a version control system than those from other disciplines [*χ*^2^(2, *N* = 184 = 16.4),  *p* < 0 .05] (Fig. 7D).

#### Sharing format and platform

**Figure 8:**
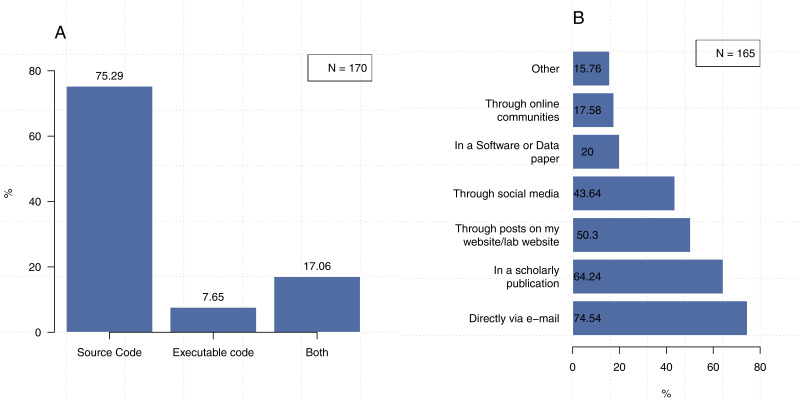
Methods and formats for sharing software. Note that the second question could be answered with more than one response. (A) In what format do you typically share your code? (B) How do you tell people about the code or software you’ve shared?

We asked our participants about how they share their code and found that 75.3% of 170 participants share their software in the form of source code, 7.6% share executables only, and 17.1% share both formats (Fig. 8A).

As shown in Fig. 8B, participants indicated that they share their software through a variety of channels, with the most common being direct communication (e.g., e-mail), which was selected by 74.5% of the participants. The figure shows that 64.2% of the time our participants make their code available through scholarly publications, 50.3% through posts on my website/lab website, and 43.6% through social media platforms. The participants who indicated that they use methods other than those listed in our survey generally responded that they do so using platforms such as GitHub or the Open Science Framework. A few researchers mentioned that they save their code along with the dataset in their institutional repository, while four participants indicated that they publicize their code via conferences.

### Preserving research software

Preserving and maintaining research software present multiple challenges for researchers and institutions (Chassanoff et al., 2018). The complex nature of software makes it hard to decide what to preserve to enable the re-use of software in the future (Rios, 2017). To understand the preservation practices around research software, we asked a variety of questions about preserving research software: Do you take any steps to ensure that your code or software is preserved over the long term; how long do you typically save your code or software; and where do you save your code or software so that it is preserved over the long term?

While research software is a building block for ensuring reproducibility, we found that 39.9% of participants (*N* = 183) indicated that they do not prepare their code for long-term preservation. We investigate how long and where researchers preserve their research software in the following subsections.

#### How long do you typically save your code or software?

**Figure 9:**
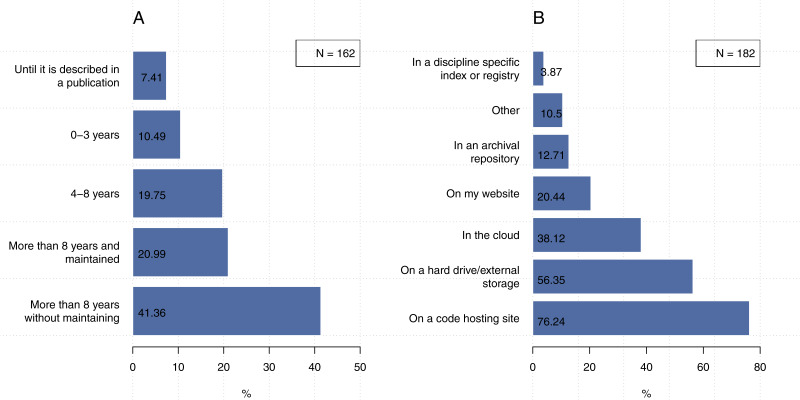
Less than half of the participants preserve their code for more than eight years without maintaining and 76.2% of them use code hosting site (e.g., GitHub) for preserving their code. Note that the second question could be answered with more than one choice. (A) How long do you typically save your code or software? (B) Where do you save your code or software so that it is preserved over the long term?

Figure 9A shows that 41.4% out of 162 participants indicated that they preserve their code for more than eight years, but generally not in a way that maintains its use. In contrast, 7.4% (*N* = 162) of participants keep their code until it is described in a publication, poster, or presentation. We found 10.5% out of 162 researchers tend to keep their codes for 3 years or less and 19.8% tend to keep their codes for 4–8 year. Only 21.0% out of 162 researchers tend to keep their codes for 8 years or more with maintaining their codes for future access and use.

####  Where do you save your code or software so that it is preserved over the long term?

In terms of where our participants preserve their code, Fig. 9B shows that code hosting sites (e.g., GitHub) was the most popular approach (76.2%), followed by hard drives or external storage (56.4%), then cloud-based facilities (38.1%). We found that 20.4% of participants indicated they use their own website to preserve their code and only 12.7% of our participants indicated that they use archival repositories (e.g., figshare). The participants who indicated “other” responses mentioned that they use a backup system of their lab, organization archive (e.g., University server), their own PC, language package registry (CRAN, PyPi or similar), Internal SVN repository, or project specific websites.

We asked participants to define *sharing* and *preserving* in their own words “please describe, in your own words, how you define “sharing” and “preserving” in the context of your code or software.” Their responses generally indicated that they make a distinction between the two concepts. For example, one participant stated succinctly, “sharing is making code available to others, in a readily usable form. Preserving is ensuring to the extent practical that the code will be usable as far into the future as possible.” However, several responses indicated that participants did not necessarily regard preservation as an active process that continues even after the conclusion of a particular project (e.g., “sharing means giving access to my code to someone else. Preserving means placing my code somewhere where it can remain and I will not delete it to save room or lose it when I switch computers or suffer a hard drive failure.” In contrast, other responses indicated that participants were aware that preservation is important for the purpose of reuse and had a knowledge of preservation tools. For example, one researcher defined preserving software as, “branching so that code remains compatible with different versions of overarching libraries (in my case) or with new coding standards and compilers” and another stated “Preserving should be done via a system like LOCKSS (https://www.lockss.org/)... Sharing can be done via the web, but must include a license so that recipients know about their rights.”

## Discussion

Scholars throughout the humanities and sciences depend on software for a wide variety of purposes, including the collection, analysis, and visualization of data (Borgman, Wallis & Mayernik, 2012; Hey, Tansley & Tolle, 2009). Though ubiquitous, software presents significant challenges to efforts aimed at ensuring reproducibility. Our results demonstrate that researchers not only create and use software for a wide variety of purposes, but also that their software-related practices are often not completely in line with those associated with reproducibility. In particular, our results demonstrate that, while scholars often save their software for long periods of time, many do not actively preserve or maintain it. This perspective is perhaps best encapsulated by one of our participants who, when completing our open response question about the definition of sharing and preserving software, wrote “Sharing means making it publicly available on GitHub. Preserving means leaving it on GitHub”. We share this anecdote not to criticize our participants or their practices, but to illustrate the outstanding need for support services related to software.

In the broader scholarly communications space, there are several prominent frameworks that relate to the reproducibility of scholarly outputs. As part of an effort to advance data as a “first class” research product, the FAIR (Findable, Accessible, Interoperable, and Reusable) guidelines provide a measurable set of principles related to the management and sharing of research data (Wilkinson et al., 2016). The FAIR principles are general enough that they can, with some modification, also be applied to software (Jimenez et al., 2017). At the level of scholarly publications, the TOP (Transparency and Openness Promotion) guidelines (Nosek et al., 2015) address citation standards and the availability of research materials including data and software. A supplement to TOP, the Reproducibility Enhancement Principles (REP) (Stodden et al., 2016) specifically targets disclosure issues related to computation and software and recommends that researchers share not only the data that underlies a set of findings, but also the software, workflows, and details related to the computational environment that produced them. However, both the TOP and REP address software are focused on scholarly publications and our results support previous research indicating that software mostly exists outside of the publication-based reputation economy of science. Therefore, while the disclosure of computational methods is important for establishing reproducibility, education-based approaches that provide guidance about software before the point it is cited in a scholarly publication are also necessary.

The majority of our participants indicated that they view code or software as a “first class” research product, that should be assessed, valued, and shared in the same way as a journal article. However, our results also indicate that there remains a significant gap between this perception and actual practice. The fact that our participants indicated that they create and use software in a wide variety of forms and for a wide variety of purposes demonstrates the significant technical challenges inherent in ensuring computational reproducibility. In contrast, the lack of active preservation and tendency to share software outside traditional (and measurable) scholarly communications channels displayed by our sample demonstrates the social and behavioral challenges. A significant difficulty in ensuring computational reproducibility is that researchers oftentimes do not treat their software as a “first class” research product. These findings reinforce the need for programs to train researchers on how to maintain their code in the active phase of their research.

At present, there are a number of initiatives focused on addressing the preservation and reproducibility of software. In the United States, the Software Preservation Network (SPN) (Meyerson et al., 2017) represents an effort to coordinate efforts to ensure the long-term access to software. The focus of SPN is generally on cultural heritage software rather than research software, but their work delineating issues related to metadata, governance, and technical infrastructure has substantial overlap with what is required for research software. In the United Kingdom, the Software Sustainability Institute trains researchers on how to develop better software and make better use of the supporting infrastructure (Crouch et al., 2013). Befitting the necessity of training and preservation indicated by our study, a similar effort, the US Software Sustainability Initiative was recently awarded funding by the National Science Foundation (NSF Award #1743188). While it is likely not possible for academic institutions to offer support services that cover the broad range of programming languages and applications described in our survey results, collaborating with such groups to create guidance and best practice recommendations could be a feasible first step in engaging with researchers about their software and code in the same manner as many research data management (RDM) initiatives now engage with them about their data. While research stakeholders including academic institutions, publishers, and funders have an interest in tackling issues of computational reproducibility in order to ensure the integrity of the research process, our results demonstrate the complexity of doing so. One participant summed up why their code could not be made re-usable: “Most of my coding is project specific and not reusable between projects because the datasets I encounter are very variable. I typically only generate packages for tasks such as getting data from a database (e.g., PubMed) and keeping RMarkdown templates in an orderly way.”

## Conclusions and Future Work

In this paper, we introduced the results of surveying researchers across different institutions on software usage, sharing, and preservation. We also checked the practices used to manage software for ensuring reproducibility and integrity of the scientific research. Our results point to several interesting trends including the widespread of writing source code and of using source code written by others, the variety of programming languages used and the lack of consistency even within the same lab or research group, the use of open source software over commercial software, and the adoption of some practices assure computational reproducibility, such as adding comments and documentation to code, but not others, specifically the general lack of active preservation. We hope our results and our survey instrument will help service providers to assess and deliver the right materials, tools, and systems to help researchers to manage their code and ensure computational reproducibility.

The present study was designed to capture a broad picture of how researchers use and share their software. For this reason, we were not able to provide a particularly granular picture of how individual practices relate to reproducible science outcomes. For example, while the majority of our participants responded that they include comments in their source code and generate documentation for their software, we were not able to make any judgment about whether or not the contents of these comments and documentation are sufficient to ensure reproducibility. Follow up research is needed in order to gain a more nuanced understanding of how processes related to the creation and use of research software relate to reproducibility. Though our sample included participants from a range of research areas and institutions, it is also likely that a more in-depth analysis of the activities of researchers affiliated with individual disciplines and institutions would reveal trends that would be informative for local research service providers. Therefore, we reiterate our hope that future research will use or adapt our survey instrument and expand upon our findings. Despite these limitations, our results indicate several potential directions for future library services centered on helping researchers create, use, and share their software and assure computational reproducibility.

##  Supplemental Information

10.7717/peerj-cs.163/supp-1 Supplemental Information 1 Understanding researcher needs and values related to software questionnaire and consent form

## Additional Information and Declarations

### Data Availability

The following information was supplied regarding data availability: Alnoamany, Yasmin; Borghi, John (2018), Data: Researcher Perspectives on the Use and Sharing of Software, v3, UC Berkeley Dash, Dataset, https://doi.org/10.6078/D1HM2W.
