# Supplementary material for: Towards computational reproducibility: researcher perspectives on the use and sharing of software
Source: PeerJ Comput Sci. 2018 Sep 17;4:e163. doi: 10.7717/peerj-cs.163 (PMC7924683; doi:10.7717/peerj-cs.163)
Supplement: Supplemental Information 1 [file peerj-cs-04-163-s001.pdf]

University of California at Berkeley
Consent to Participate in Research

Understanding researcher needs and values related to software

Introduction and Purpose
We are investigating researcher needs and values related to software and computer code. Your responses may help us and other organizations develop services that meet the needs of the research community.

Procedures
If you agree to participate in our study, we will ask you to complete a survey that includes questions about how you create, use, and share computer code in the course of your research. It should take about 10-20 minutes to complete.

Benefits
There is no direct benefit to you from taking part in this study.

Risks/Discomforts
No potentially sensitive items are included in our survey, and therefore we do not anticipate that your participation poses any personal or professional risk.

Confidentiality
The information obtained by this study will be used only for research purposes and in ways that will not reveal who you are. Federal or state laws may require us to show information to university or government officials (or sponsors) who are responsible for monitoring the safety of this study. Your responses will be recorded anonymously. Any personal information that could identify you will be removed or changed before files are shared in any way (including with other researchers) or the results of this study are made public.

Compensation
You will not be paid for taking part in this study.

Rights

Participation in research is completely voluntary. You are free to decline to take part in this study. You can decline to answer any survey questions and are free to stop taking part in the study at any time. Whether or not you choose to participate, to answer any particular questions, or continue participating in the project, there will be no penalty to you or loss of benefits to which you are otherwise entitled.

Questions
If you have any questions about this study, please contact Yasmin AlNoamany at yasminal@berkeley.edu

If you have any questions about your rights or treatment as a research participant in this study, please contact the University of California at Berkeley's Committee for Protection of Human Subjects at 510-642-7461, or e-mail subjects@berkeley.edu

If you agree to take part in the research, please click the "Next" button below.

Demographic Questions

Thank you for agreeing to participate in our study!

Before we ask about how you use, share, and value computer code and software, we have a few questions about who you are and what you do. This information will help us contextualize your responses to our other survey questions.

1. How old are you (in years)?_____

2. What is the highest degree you hold?
    - ○ High school
    - ○ Associate
    - ○ Bachelors
    - ○ Masters
    - ○ Professional degree
    - ○ Doctorate

3. What is your professional title or role?
    - ○ Principal Investigator
    - ○ Research Faculty
    - ○ Staff
    - ○ Postdoc
    - ○ Graduate Student
    - ○ Undergraduate Student
    - ○ Research Assistant
    - ○ Other _____

4. What is your field or discipline (e.g. Psychology, Computer Science)? _____

5. What is your employer/institutional affiliation (e.g. UC Berkeley)? _____

6. Which of the following best describes your employer/institution?
    - ○ Academic: Research Focused
    - ○ Academic: Teaching Focused
    - ○ Academic: Medical School
    - ○ Government
    - ○ Nonprofit
    - ○ Commercial
    - ○ Other _____

7. Who funds your research or work (e.g. NIH, Gates Foundation)? _____

This survey is composed of three sections, each addressing a different aspect of what researchers need and value when it comes to code and software.

Section 1: Characteristics of Your Code and Software

Section 2: Sharing Your Code and Software

Section 3: Valuing Code and Software

Because some of the questions in these sections contain words with multiple or ambiguous meanings, we have provided a list of definitions.

Please feel free to refer back to this list at any point.

Source Code (aka Code): A collection of computer instructions, written using a human-readable programming language like Python, C++, or SQL.

Executable: A file that tells a computer to execute (or run) a program. Executable files are also sometimes called binaries and are often saved in .exe format.

Commercial Software: Software that is produced for sale. Commercial software is generally only available in executable form and the software's publisher generally retains copyright over the source code. Examples of commercial software include Microsoft Office, SPSS, and Stata.

Open source software: Software with source code that anyone can inspect, modify, and distribute for any purpose. Examples of open source software include LibreOffice, PSPP, and JASP.

Comments: Human-readable explanations or annotations <u>within</u> computer code. Comments can be used to describe code or explain the programmer's intent.

Documentation: Texts or illustrations that explain how to use a piece of code or software. Documentation could include README files, FAQs, and usage examples.

Section 1: Characteristics of Your Code and Software

The following questions address the characteristics of the code and/or software you use as part of your research.

1. Have you received training in coding conventions or best practices?
   - ○ Yes
   - ○ No

2. Do you write source code in the course of your research?
   - ○ Yes
   - ○ No
   - ○ I don’t know

3. Do you use source code written by others (i.e. code not compiled into an executable)?
   - ○ Yes
   - ○ No
   - ○ I don’t know

4. Do you create executables in the course of your research?
   - ○ Yes
   - ○ No
   - ○ I don’t know

5. Do you use executables created by others?
   - ○ Yes
   - ○ No
   - ○ I don’t know

6. Do you use commercial software in the course of your research?
   - ○ Yes
   - ○ No
   - ○ I don’t know

7. Do you use open source software in the course of your research?
   - ○ Yes
   - ○ No
   - ○ I don’t know

8. How do you use code or software? [Please check all that apply.]
   - ○ To automate part of the research process
   - ○ To collect data
   - ○ To analyze data
   - ○ To clean/organize data
   - ○ To visualize data
   - ○ Other _____
   - ○ Not applicable

9. Have you ever repurposed your code or software (i.e. using it for a project other than the one for which it was originally created)?
    - Yes
    - No
    - I don't know
    - Not applicable

10. Which programming language(s) do you use for writing code? [Click all that apply.]
    - C
    - C#
    - C++
    - Java
    - Javascript
    - Matlab
    - PERL
    - PHP
    - Python
    - R
    - Ruby
    - SQL
    - Swift
    - Other _____
    - Not applicable

11. Does everyone in your lab or research group write code using the same programming language(s)?
    - Yes
    - No
    - I don't know
    - Not applicable

12. Do you include comments in your code?
    - Yes
    - No
    - I don't know
    - Not applicable

13. Do you generate documentation for your code?
    - Yes
    - No
    - I don't know
    - Not applicable

14. Do you write code collaboratively (i.e. with another person or multiple people)?
    - Yes
    - No
    - I don't know
    - Not applicable

15. Do you write code using a “notebook” application (e.g. IPython, Jupyter, Zeppelin, Beaker)?
    - ○ Yes
    - ○ No
    - ○ I don’t know
    - ○ Not applicable

16. Would another person in your field be able to use your code or software to replicate your research given your degree of annotation and documentation?
    - ○ Yes
    - ○ No
    - ○ I don’t know
    - ○ Not applicable

17. [Open Response Question] Please describe, in your own words, how you use software or code in your research.

    ____
    ____
    ____
    ____
    ____
    ____
    ____
    ____

Section 2: Sharing Your Code and Software

The following questions ask about how you share the code and/or software you use as part of your research and how you find the code and/or software shared by others.

1. Are you aware of any community standards in your field or discipline about sharing code or software?
    - ○ Yes
    - ○ No

2. Do you share the code or software created as part of your research?
    - ○ Yes
    - ○ No
    - ○ I don’t know
    - ○ Not applicable

3. Is there any reason your code or software could not be shared?
    - ○ Yes [Please describe] _____
    - ○ No
    - ○ I don’t know
    - ○ Not applicable

4. How do you tell people about the code or software you’ve shared? [Click all that apply]
    - ○ I communicate with them directly (e.g. e-mail)
    - ○ I describe my code in a “traditional” scholarly publication (e.g. a research paper)
    - ○ I describe my code in a “Software” or “Data” paper.
    - ○ Through posts on my website/lab website
    - ○ Through social media (e.g. Twitter)
    - ○ Through online communities (e.g. Stack Overflow)
    - ○ Other [Please specify] ____
    - ○ Not applicable

5. In what format do you typically share your code? [Click all that apply]
    - ○ Source Code
    - ○ Executable Code
    - ○ Other _____

6. If you make a change to your code, do you share a new version?
    - ○ Yes
    - ○ No
    - ○ I don’t know
    - ○ Not applicable

7. Do you use a version control system (e.g. Git, SVN)?
    ○ Yes
    ○ No
    ○ I don't know
    ○ Not applicable

8. When you share your code or software, do you share it alongside related files (e.g. datasets)?
    ○ Yes [Please describe] _____
    ○ No
    ○ I don't know
    ○ Not applicable

9. When you share your code or software, do you provide information about dependencies?
    ○ Yes
    ○ No
    ○ I don't know
    ○ Not applicable

10. How long do you typically save your code or software?
    ○ Until it is described in a publication, poster, or presentation.
    ○ 0-3 years
    ○ 4-8 years
    ○ More than 8 years (and maintained so it is always usable and accessible)
    ○ More than 8 years (but in a format that may become obsolete/inaccessible)
    ○ Not applicable

11. Do you take any steps to ensure that your code or software is preserved over the long term?
    ○ Yes [Please describe] _____
    ○ No
    ○ I don't know
    ○ Not applicable

12. Where do you save your code or software so that it is preserved over the long term?
    ○ On a code hosting site (e.g. GitHub)
    ○ In an archival repository (e.g. Figshare)
    ○ On my website
    ○ On a hard drive/external storage
    ○ In the cloud
    ○ In a discipline specific index or registry (e.g. ASCL)
    ○ Other _____
    ○ Not applicable

13. Do you integrate code or software written by others into your own?
    ○ Yes
    ○ No
    ○ I don't know
    ○ Not applicable

14. How do you find code or software written by others? [Click all that apply]
    - Conversations with collaborators
    - Reading the scholarly literature
    - Using a search engine (e.g. Google)
    - Using social media (e.g. Twitter)
    - Searching online communities (e.g. Stack Overflow)
    - Searching code hosting sites (e.g. GitHub)
    - Other [Please specify] ____
    - Not applicable

15. Where do you typically find code or software written by others? [Click all that apply]
    - It's given to me directly by my collaborators
    - It's given to me directly by the authors
    - Hosted alongside a scholarly publication.
    - In a "Data" or "Software" paper.
    - On the author's website.
    - On a code hosting site (e.g. GitHub)
    - In an archival repository (e.g. Figshare)
    - Other [Please specify] ____
    - Not applicable

16. Do you typically encounter comments within the code you find?
    - Yes
    - No
    - I don't know
    - Not applicable

17. Do you typically encounter documentation for using the code you find?
    - Yes
    - No
    - I don't know
    - Not applicable

18. [Open Response Question] Please describe, in your own words, how you define "sharing" and "preserving" in the context of your code or software.

    ____
    ____
    ____
    ____
    ____
    ____
    ____
    ____

Section 3: Valuing Code and Software

The following questions ask about how you credit the code and/or software you use as part of your research and how you'd like the code and/or software you create to be credited by others.

1. Are you aware of any best practice guidelines (in your discipline or in general) related to assigning credit to code or software?
    - Yes
    - No

2. Have you ever cited a piece of code or software in a scholarly work (e.g. a paper, poster, or presentation)?
    - Yes [Please describe] _____
    - No
    - I don't know
    - Not applicable

3. How have you cited a piece of code or software? [Click all that apply]
    - Using a standard citation format (e.g. APA style).
    - By citing the publication describing the software or code.
    - By citing the user manual for the software or code.
    - By citing the software or code as if it were an instrument.
    - By citing the project name or website.
    - By providing an in-text link or URL.
    - By mentioning the software's name (with version number).
    - By mentioning the software's name (without version number).
    - By providing the information given in the code's "Citation" file.
    - Other [Please specify] ____
    - I have never cited a piece of code or software.

4. How have you given credit to the creator(s) of a piece of code or software? [Click all that apply]
    - By mentioning author(s) by name(s) (e.g. Jane Smith).
    - By mentioning the working group (e.g. "The R Project Team").
    - By mentioning the commercial entity that released the code (e.g. Mathworks).
    - Other [Please specify] ____
    - I have never given credit to the creators of a piece of code or software.

5. Have you used code or software for which the creators have provided specific guidelines about how they'd like it to be credited or cited?
    - Yes
    - No
    - I don't know
    - Not applicable

6. Has code or software you've written entirely yourself been cited in a scholarly work (e.g. a paper, poster, or presentation)?
    - ○ Yes
    - ○ No
    - ○ I don't know
    - ○ Not applicable

7. Has code you've contributed to been cited in a scholarly work (e.g. a paper, poster, or presentation)?
    - ○ Yes
    - ○ No
    - ○ I don't know
    - ○ Not applicable

8. Have you ever provided specific instructions as to how you would like your code or software to be cited?
    - ○ Yes
    - ○ No
    - ○ I don't know
    - ○ Not applicable

9. What type of credit would you like to receive when others make use of code you have written entirely yourself? [Click all that apply]
    - ○ A citation using a standard citation format (e.g. APA style).
    - ○ A citation for the publication describing my code or software.
    - ○ A citation for the user manual for my code or software.
    - ○ A citation that describes my code or software as an instrument.
    - ○ A mention/reference to the project name or website.
    - ○ An in-text link or URL.
    - ○ A mention of my code or software's name (including version number)
    - ○ A mention of my code or software's name (without version number)
    - ○ Whatever I have specified in my code or software's "Citation" file.
    - ○ Other [Please specify] ____
    - ○ Not applicable.

10. What type of credit would you want to receive when others make use of code or software you have contributed to or modified?
    - A citation using a standard citation format (e.g. APA style).
    - A citation for the publication describing my code or software.
    - A citation for the user manual for my code or software.
    - A citation that describes my code or software as an instrument.
    - A mention to the project name or website.
    - An in-text link or URL.
    - A mention of my code or software's name (including version number)
    - A mention of my code or software's name (without version number)
    - Whatever I have specified in my code or software's "Citation" file.
    - Other [Please specify] ____
    - Not applicable.

11. How do you measure the reach of a piece of code or software that you've made public or shared? [Click all that apply]
    - Views
    - Downloads
    - Citations
    - User ratings (e.g., Github stars)
    - Contributions to a project or repository (e.g. Forks)
    - Social media mentions
    - Other [Please specify] ____
    - Not applicable

12. On what basis do you assess the quality of a piece of code or software? [Click all that apply]
    - Ease of use
    - Effectiveness at accomplishing a desired task
    - Quality and richness of associated documentation
    - Quality of associated publication(s)
    - Reputation of the repository
    - Reputation of the creator(s)
    - Amount of reuse by others
    - Other [Please specify] ____

13. Do you regard code or software as a "first class" research product (i.e. a product that should be assessed, valued, and shared in the same way as a journal article)?
    - Yes [Please describe] _____
    - No
    - I don't know
    - Not applicable

14. [Open Response Question] Please describe, in your own words, how you assess the value of the code and software you create or use.

____
____
____
____
____
____
____

The survey is now complete. Thank you for your participation!

If you have any questions about this research, please contact at Yasmin AlNoamany at yasminal@berkeley.edu
